# Effects of Pharmacotherapy for the Treatment of Obesity in an Urban, Safety-Net Population

**DOI:** 10.7759/cureus.47922

**Published:** 2023-10-29

**Authors:** Eric K Kim, Nancy K Hills, Zoe Cheng, Chloe Tucker, Maria Gutierrez, Diana Alba, Elizabeth Murphy, Sarah Kim

**Affiliations:** 1 Department of Otolaryngology - Head and Neck Surgery, University of California San Francisco, San Francisco, USA; 2 Department of Neurology, University of California San Francisco, San Francisco, USA; 3 Division of Endocrinology, Diabetes and Metabolism, Department of Medicine, Zuckerberg San Francisco General Hospital and Trauma Center, San Francisco, USA

**Keywords:** topiramate, phentermine, glp-1, pharmacotherapy, obesity, weight loss

## Abstract

Objective: To evaluate the effect of common weight loss pharmacotherapies among low-income, racially diverse adult patients at an urban safety-net weight management clinic.

Methods: Our retrospective review from 2015 to 2019 examined patients who took either GLP-1 analog (GL) or phentermine/topiramate (PT) for ≥90 days and patients who exclusively pursued non-pharmacologic treatment for comparison. Changes in weight, blood pressure, and hemoglobin A1c at 1-year follow-up were reported.

Results: We analyzed 22 GL and 26 PT patients and included 40 patients who pursued only lifestyle modifications (LM). All three groups achieved significant weight loss at one year: GL -3.69 (interquartile range (IQR): -11.0, -1.77) kg (p=0.0004), PT -7.01 (IQR: -13.4, -1.45) kg (p<0.001), and LM -3.01 (IQR: -6.81, 1.13) kg (p=0.005). There was no significant difference in the median weight loss (p=0.11) between the three groups. We observed no significant changes in systolic blood pressure but saw a significant change of -0.75 in hemoglobin A1c (IQR: -1.35, -0.25) (p=0.01) among patients with diabetes in the GL group.

Conclusions: Our real-world applications of GLP-1 and phentermine/topiramate suggest that both are effective weight loss medication regimens in low-socioeconomic status patients.

## Introduction

Obesity is a highly prevalent disease associated with serious comorbidities, such as cardiovascular disease, type 2 diabetes, and cancer. It is also a chronic disease that disproportionately affects low-income and racial minority groups, a phenomenon that has been attributed to a complex interplay between factors including race, education, and socioeconomic status [1,2]. San Francisco, California, exemplifies this health phenomenon. In 2012, the rate of obesity in San Francisco was 17%, lower than the state average of 23%. However, among residents of color, 33% of the city’s African-Americans and 57% of the county’s Latino/Hispanic population had obesity [3].

Currently, recommended obesity treatments include behavioral and dietary lifestyle changes, bariatric surgery, and long-term pharmacologic treatments [4]. Of the medical therapies, GLP-1 analogs (GL) and phentermine/topiramate (PT) have proven to be modestly effective for weight reduction and associated with improved metabolic and cardiovascular health [5,6]. These benefits also have to be balanced with potential complications of long-term use of these medications, such as gastrointestinal issues, headaches, and nasopharyngitis [7].

Studies on obesity treatment for low-income and racial minority populations are mainly limited to diet, exercise, and behavioral counseling. One study in an urban safety net population showed that a "toolbox” consisting of partial meal replacements, recreation center vouchers, medications, and group classes resulted in ≥5% weight loss in 25% of subjects [8]. In a randomized controlled trial (RCT) of Black women who were either overweight or had class 1 obesity, the intervention group (tailored behavior change goals, weekly self-monitoring, monthly counseling calls, skills training materials, and a gym membership) achieved a greater 12-month weight reduction than the control group (-1.0 kg vs. +0.5 kg, p=0.04). A cluster RCT of 803 underserved adults with obesity also showed that those who underwent a high-intensity, lifestyle-based treatment program achieved a greater percent weight loss at 24 months, with a mean between-group difference of -4.51 percentage points (p<0.001) [9]. Finally, residents of rural communities randomized to intensive-lifestyle interventions experienced a greater weight loss than their usual-care control counterparts (-2.6 kg vs. -0.4 kg, p<0.01) [10]. These studies demonstrate that lifestyle-based management of obesity is effective, yet there is limited data on the effects of common weight loss pharmacotherapies in underserved patient populations.

To address this gap, we evaluated the real-world efficacy of GLP-1 analogs and phentermine/topiramate on weight loss as well as obesity-related comorbidities at the Zuckerberg San Francisco General Hospital (ZSFG) Weight Management Clinic (WMC), a safety net clinic housed under the San Francisco Department of Public Health.

## Materials and methods

Study population

The ZSFG WMC was established in 2015 to serve patients with a body mass index (BMI) of 40+ or BMI of 35+ and obesity-related comorbidity (e.g., hypertension, diabetes mellitus, dyslipidemia, and sleep apnea). Patients are referred to the WMC from a network of safety-net clinics that take care of underinsured and uninsured patients across the city. The WMC team consists of three endocrinologists and a dietitian, offering one-on-one consultations as well as monthly group classes on nutrition and exercise. Patients are also given the opportunity to pursue any combination of three options: pharmacotherapy (primarily GLP-1 analogs and/or phentermine/topiramate), lifestyle counseling (including the option to enroll in a Diabetes Prevention Program (DPP) free of cost), and referral to bariatric surgery. For lifestyle modifications (LMs), patients were generally counseled about diet (e.g., minimizing sugar-sweetened beverages, ultra-processed snack foods, and fried foods, avoiding eating out, and increasing intake of fruits, vegetables, and whole grains) and activity (exercising at least 30 minutes per day).

Phentermine and topiramate were prescribed separately with a phentermine dose of 15 mg daily and topiramate of 50 mg twice a day. A standard titration of phentermine dosage was not used. Patients paid out of pocket for phentermine and had access to a coupon for 15 mg of phentermine, so it was started at the maximum dose. GLP-1 used was predominantly liraglutide 1.8 mg daily. Due to insurance and financial constraints, the use of GLP-1 analogs was mainly limited to brands and doses indicated for type 2 diabetes.

We conducted a retrospective analysis of all patients seen at the WMC from November 2015 to June 2019. Demographic information (age, gender, sex, race/ethnicity, and primary language) and relevant medical history were collected by chart review. The patient was determined to have hypertension, hyperlipidemia, and depression/anxiety if stated in the medical records or if the patient was prescribed antihypertensive, statin, or antidepressant therapy, respectively. The presence of pre-diabetes was determined by an HbA1c level of 5.6-6.4% in the absence of glucose-lowering therapy. The presence of type 2 diabetes was determined from the medical record and confirmed by either an HbA1c >6.4% or a prescription for glucose-lowering therapy.

Inclusion and exclusion criteria

Patients with severe obesity (BMI of ≥35) seen at the ZSFG WMC who took either GLP-1 analog or phentermine/topiramate for ≥90 days in addition to LMs and had a one-year follow-up were included. Dates of the first and last prescriptions on the electronic prescription log were used to determine medication usage duration. Clinic patients who pursued only counseling and LM and had weight data one year after the initial WMC visit were included for comparison. For individuals who ultimately received bariatric surgery, their data were included if they had a one-year follow-up prior to surgery. Those who did not follow up at the WMC after the initial visit were excluded. Patients who took both GLP-1 analogs and phentermine/topiramate were also excluded from the analysis.

Outcomes

The primary outcomes were weight reduction and percent body weight loss at one year. If the patient did not have a recorded weight on the exact date one year from the first visit, the weight measured closest to the one-year mark was used. The secondary outcomes included systolic and diastolic blood pressure and hemoglobin A1c (HbA1c) changes at the one-year follow-up. For HbA1c analysis, we included only patients with type 2 diabetes or pre-diabetes who had recorded HbA1c measurements within three months of the initial visit and one-year follow-up. If a patient was missing either of the two values, their data were not included. This study was reviewed and approved by the University of California, San Francisco, institutional review board (#20-31042).

Statistical analysis

Characteristics of the three groups were summarized and compared at baseline. Continuous variables, including age, BMI, blood pressure, and HbA1c, were summarized with means and standard deviations; categorical data, including sex, race/ethnicity, primary language, and prevalence of comorbidities (e.g., diabetes, hypertension, and hyperlipidemia), were summarized with frequencies and proportions. Continuous variables were compared across the three treatment groups using ANOVA, and categorical variables were compared using chi-square or Fisher’s exact tests.

Weight loss after one year (measured both by difference in kilograms and percent body weight loss) was compared within each group using the Wilcoxon signed rank test and across the three groups using the non-parametric Kruskal-Wallis test. Post-hoc pairwise comparisons of variables significant in omnibus testing were performed using Dunn’s test with the Holm multiple comparison adjustment. Reductions in blood pressure and hemoglobin A1c (in the subset of patients diagnosed with diabetes) were similarly compared across the three groups. Statistical significance was defined as p<0.05 (except when multiple comparison adjustments were made). Stata v.16 (StataCorp, College Station, TX, USA) was used for analysis.

## Results

**Figure 1:**
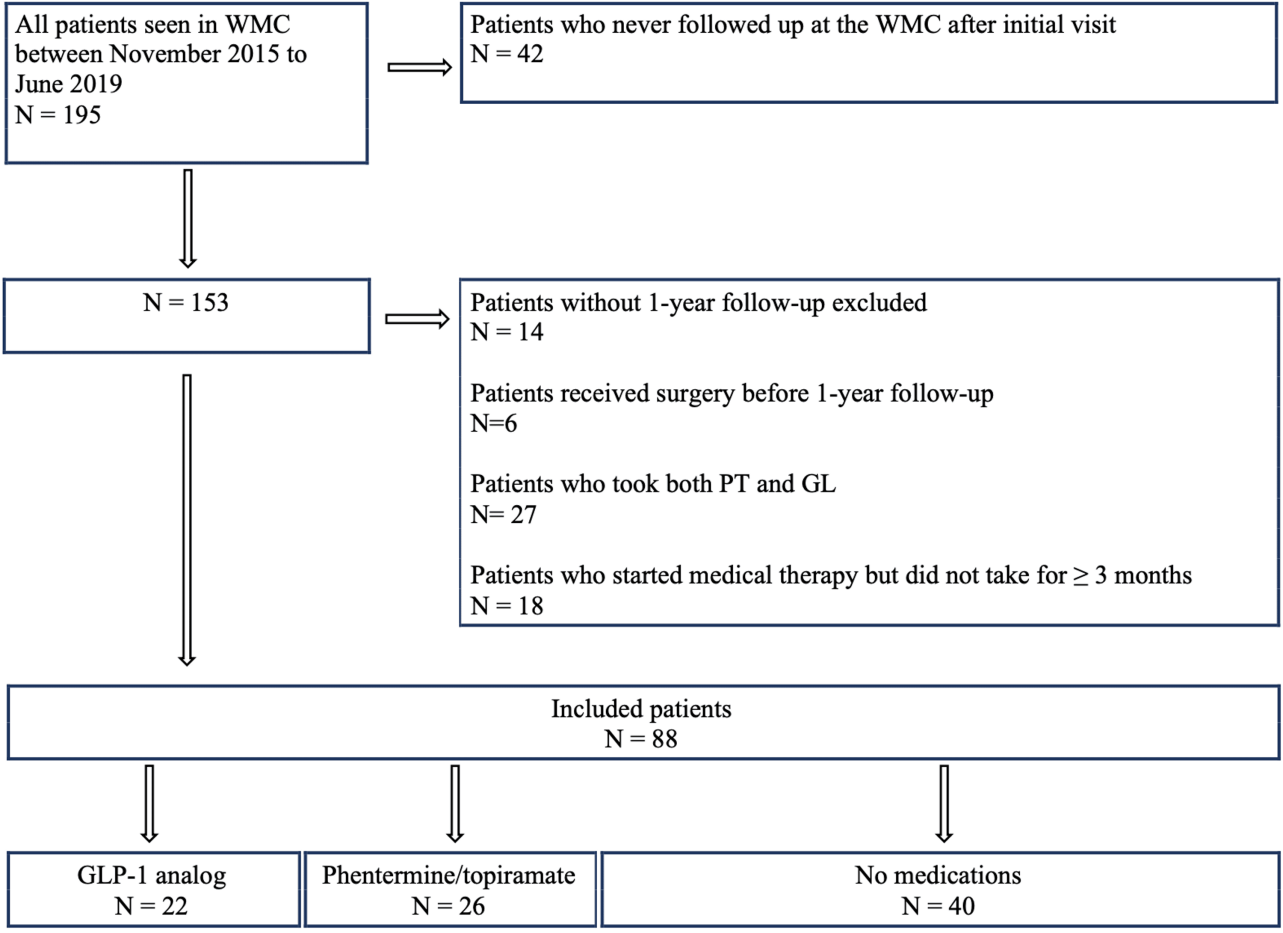
Participant flow diagram.

Of 195 consecutive patients seen at the WMC, we included 22 GL, 26 PT, and 40 LM patients (participant flow diagram shown in Figure 1). The baseline characteristics of the study population are shown in Table 1. The average age was 48 (standard deviation: 13 years), and 78% of the patients were female. Latino/Hispanic patients comprised 52% of the study population, followed by 25% white, 15% Asian/Pacific Islander, and 8% African-American. English was the primary language for 71% of participants; 28% spoke Spanish. The PT group was younger than the two other groups (p=0.006). The GL group had a higher rate of diabetes than the other two groups (77% GL vs. 12% PT vs. 25% LM, p<0.001). The PT group had a significantly lower rate of hypertension (73% GL vs. 31% PT vs. 58% LM, p=0.012) and hyperlipidemia (73% GL vs. 15% PT vs. 50% LM, p<0.001) than the other two groups.

**Table 1:** Baseline characteristics of the study population. Abbreviations: BMI = Body mass index; SD = Standard deviation. *: Statistically significant, P<0.05 by ANOVA between GLP-1 analog, Phentermine/topiramate, and No medications groups If p< 0.05 on the ANOVA, the Bonferroni adjustment was used to make pairwise comparisons (Pbon<0.0167). Superscripts (a and b) signify statistical significance. Values that share superscripts are not statistically different. $: One individual identified as biracial (Hispanic/Latino and American Indian).

Characteristics	Total (N=88)	GLP-1 analog (N=22)	Phentermine/topiramate (N=26)	Lifestyle modifications (N=40)	P-value
Sex – no. (%)					0.597
Female	69 (78)	16 (73)	22 (85)	31 (78)	
Male	19 (22)	6 (27)	4 (15)	9 (12)	
Race/ethnicity – no. (%)					0.130
White	22 (25)	6 (27)	7 (27)	9 (23)	
Hispanic	46 (52)	13 (59)^$^	15 (58)	18 (45)	
African-American	7 (8)	2 (9)	3 (11)	2 (5)	
Asian/Pacific Islander	13 (15)	1 (5)	1 (4)	11 (28)	
Primary language – no (%)					0.407
English	62 (71)	13 (59)	21 (81)	28 (70)	
Spanish	25 (28)	9 (41)	5 (19)	11 (28)	
Cantonese	1 (1)	0 (0)	0 (0)	1 (2)	
Patient details at baseline – no (%)					
Mean age±SD, yr	48±13	54±9^a^	43±13^b^	50±12^a^	0.006^†^
Mean BMI±SD, kg/m^2^	45±7	45±7	45±7	47±6	0.562
Mean weight±SD, kg	117±23	118±19	121±28	123±23	0.724
Mean SBP±SD, mmHg	119±15	122±16	125±12	125±17	0.734
Mean DBP±SD, mmHg	74±8	78±10	79±7	76±8	0.280
Comorbidities – no (%)					
Pre-diabetes	23 (26)	3 (14)	8 (31)	12 (30)	0.304
Diabetes mellitus	30 (34)	17 (77)^a^	3 (12)^b^	10 (25)^b^	<0.001*
Hypertension	37 (53)	16 (73)^a^	8 (31)^b^	23 (58)^a^	0.012*
Anxiety/Depression	51 (58)	11 (50)	13 (50)	27 (68)	0.254
Hyperlipidemia	40 (45)	16 (73)^a^	4 (15)^b^	20 (50)^a^	<0.001*

The median duration of medication usage was not statistically different between the GL and PT groups, with a median of 355 days (interquartile range (IQR): 209-514) days. Eighty-one percent of the included patients took the medications for 180 days, and 65% of the patients took medications for at least 300 days.

Weight

The mean BMI for the entire cohort was 45±7 kg/m^2^. There was no difference in baseline BMIs across the three groups. All three groups achieved significant weight reduction from the initial clinic visit and the one-year follow-up. In the GL group, there was a median difference of -3.69 (IQR: -11.0, -1.77) kg (p=0.0004), resulting in a median percent body weight change of -3.1 (IQR: -9.1, -1.6) %. In the PT group, there was a median difference of -7.01 (IQR: -13.4, -1.45) kg (p<0.001), resulting in a median percent body weight change of -5.8 (IQR: -9.2, -1.3) %. In the LM group, there was a median difference of -3.01 (IQR: -6.81, 1.13) kg (p=0.005) resulting in a median percent body weight change of -2.3% (IQR: -5.85, 1.05). There was no significant difference in the median weight loss (p=0.11) or median percent body weight loss (p=0.10) across the three groups.

**Figure 2:**
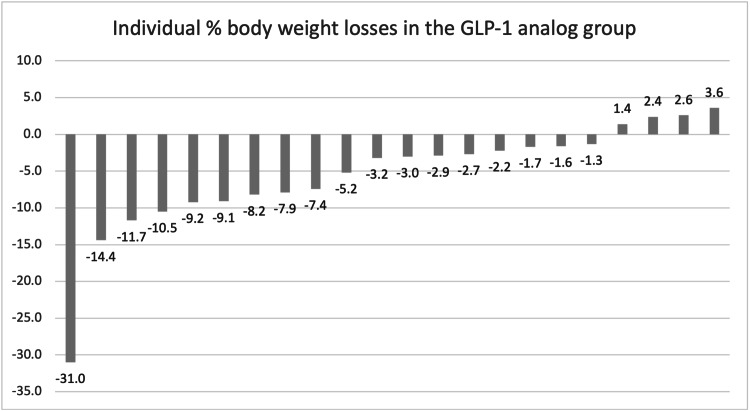
Individual % body weight loss in the GLP-1 analog group.

**Figure 3:**
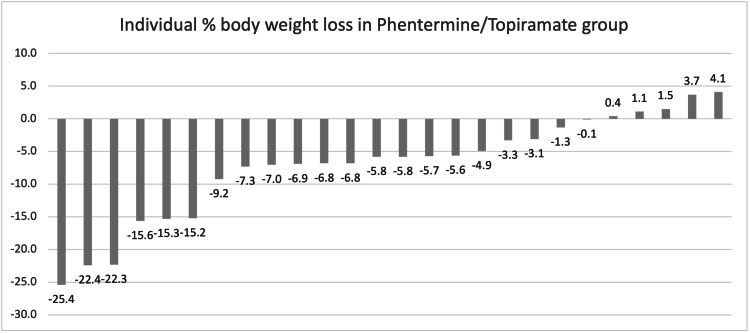
Individual % body weight loss in the phentermine/topiramate group.

**Figure 4:**
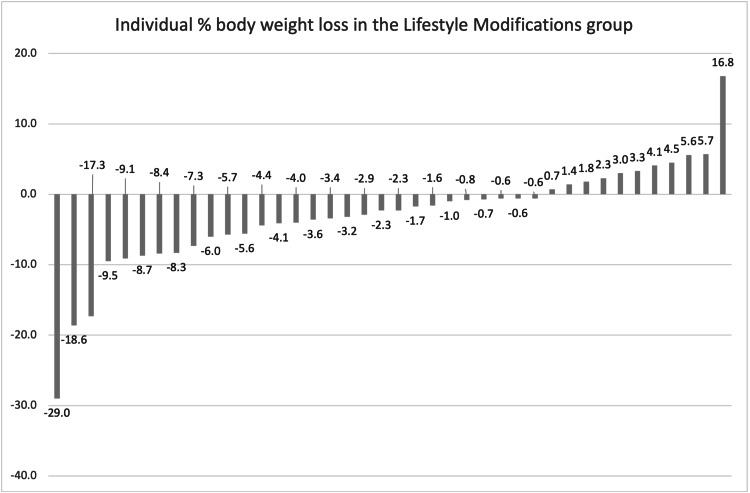
Individual % body weight loss in the lifestyle modifications group.

Overall, 43% of the entire cohort achieved the clinically relevant marker of 5% weight loss. Sixty-two percent of the PT group, 45% of the GL group, and 30% of the LM group achieved 5% weight loss. Twenty-three percent of the PT group, 18% of the GL group, and 8% of the LM group achieved 10% weight loss. Twenty-three percent of the PT group, 8% of the LM group, and 5% of the GL group achieved 15% weight loss. All results are demonstrated in Table 2. Individual weight losses for each of the groups have been graphed in Figures 2-4.

**Table 2:** Changes in weights, blood pressure, and hemoglobin A1c: comparing baseline vs. one-year follow-up. ** Total N=36 (LM=14, GLP=16, LM=6); pairwise tests using Holm's multiple comparison adjustment indicate that the LM and GLP groups are significantly different (<=0.005).

Change at 1 year	Lifestyle Modification (N=40)		GLP-1 Analog (N=22)		Phentermine/Topiramate (N=26)		p-value*
	Median	(IQR)	Median	(IQR)	Median	(IQR)	
Weight loss (kg)	-3.01	(-6.81, 1.13)	-3.69	(-11.0, -1.77)	-7.01	(-13.4, -1.45)	0.11
Body weight loss (%)	-2.3	(-5.85, 1.05)	-3.1	(-9.1, -1.6)	-5.8	(-9.2, -1.3)	0.09
Reduction in SBP (mm Hg)	2	(-5.5, 10)	3.5	(-12, 9)	-5	(-15, 5)	0.14
Reduction in DBP (mm Hg)	2	(-2, 7.5)	-4	(-9, 3)	-1	(-6, 5)	0.06
Reduction in hemoglobin A1c**	0.05	(-0.2, -0.3)	-0.75	(-1.35, -0.25)	-0.45	(-0.5, -0.3)	0.01

Blood pressure and HbA1c

No significant blood pressure changes were observed within any of the three groups, for either systolic or diastolic pressure, and there were no differences across the three groups. Among 16 total patients with diabetes in the GL group, HbA1c changed by -0.75 (IQR: -1.35, -0.25) p=0.01. Hemoglobin A1c did not change in the PT group (n=3) and LM group (n=11).

## Discussion

Studies have demonstrated that social determinants of health have a significant impact on obesity and account for the high prevalence of obesity. Historical and ongoing structural violence, limited access to healthy foods, lack of infrastructure for physical activity, and unsafe environments are just a few of many contributing factors to obesity faced by disadvantaged groups [11,12]. In one small prospective cohort study of 21 low-income patients in Korea, the authors found that phentermine, orlistat, and topiramate resulted in a significant reduction in weight, waist circumference, and low-density lipoprotein [13]. To the best of our knowledge, this study is the first to specifically study the effect of obesity pharmacotherapy in a U.S. urban safety net population.

Because our cohort is 52% Hispanic/Latino, it is worth noting the results of GLP-1 analogs on weight loss in clinical trials with Hispanic subjects. One RCT demonstrated that the efficacy and safety profiles of liraglutide 3.0 mg are similar between Hispanic and non-Hispanic groups [14]. In a post-hoc analysis of phase III trials, one of the trials showed that Hispanic patients achieved significant weight loss with liraglutide 1.2 mg and 1.8 mg [15]. Our results support these promising clinical trial findings and demonstrate that these medications are effective in real-world settings of a low-income, majority-Hispanic patient population.

Our findings suggest that GLP-1 analogs and phentermine/topiramate combinations are all effective weight-loss medications in a diverse safety-net population. The medications also had either neutral or beneficial effects on HbA1c and blood pressure. This was true even among patients who used PT, which has the potential to raise blood pressure. Unsurprisingly, patients who took GLP-1 analogs, which are indicated for treating diabetes, showed a significant reduction in HbA1c while losing weight. Patients who pursued only non-pharmacologic methods also showed significant weight loss, which is consistent with the literature [8-10]. We observed no significant differences in the mean weight loss between the three groups. This may indicate that our LM patients were able to lose weight through the successful implementation of lifestyle changes learned in the WMC classes and counseling sessions, which made their weight loss results comparable to those of the pharmacotherapy groups. While we noticed that a greater proportion of the PT group achieved 5% weight loss (a clinical marker linked with cardiovascular benefits [16]) than the LM group, a greater sample size is required to draw definitive conclusions regarding the superiority of one treatment.

This study has some notable limitations. First, our study is retrospective and has a small sample size, which limits the power of the study and the generalizability of our findings. Because the patient population of a safety net clinic faces various challenges to accessing and continuing care at a longitudinal clinic, our numbers are unsurprisingly low. However, we hope to demonstrate that individuals who are able to overcome these challenges and use a weight loss medication for at least a year see real benefits. Second, not all demographic information was available; for example, the exact insurance type of each subject was not collected, which restricts our understanding of the affordability of pharmacotherapies for different patients. Third, not all clinical information was available. To determine each patient’s duration of medication usage, we used the medications’ prescription dates as proxies. Because we have no actual data on the patient’s adherence to the medications, we had to assume that all patients were completely adherent. We also do not have exact data on the LMs that each subject undertook. Additionally, patients were not randomized to different treatment arms, which makes direct intergroup comparisons of weight loss challenging. Patients in the LM group did not take pharmacotherapy for a variety of reasons, including patient preferences and contraindications, and may have self-selected into this group. A final limitation is that the GLP-1 analogs used for our patients were brands and doses designated for diabetes management because liraglutide 3.0 mg was more difficult to obtain through health insurance and semaglutide 2.4 mg was not yet available. Astrup et al. showed that liraglutide 1.8 mg and 3.0 mg achieved a 5.5 kg and 7.2 kg weight loss, respectively [17]. Therefore, it is possible that our patients could have achieved greater weight loss with liraglutide 3.0 mg. However, our findings also provide evidence that dosages and brands for diabetes management can also result in significant weight loss.

## Conclusions

This longitudinal retrospective study examined the efficacy of common weight loss medical therapies in a patient population that has been often neglected in the literature but is also at the highest risk of obesity and its complications. In our cohort of low-income and racially diverse patients with severe obesity, we showed that GLP-1 analog and phentermine/topiramate combinations effectively reduce weight. Patients who pursued only lifestyle changes experienced significant weight reduction, although a smaller proportion achieved 5% weight loss compared to the phentermine/topiramate group. Given the similarity of our patient demographics to those of other safety net clinics, we believe that our results should empower providers working with underserved populations to use these agents as an efficacious adjunct therapy to lifestyle modifications.
